# Supplementary material for: Geostatistical Modeling and Heterogeneity Analysis of Tumor Molecular Landscape
Source: Cancers (Basel). 2022 Oct 25;14(21):5235. doi: 10.3390/cancers14215235 (PMC9656041; doi:10.3390/cancers14215235)
Supplement: Supplementary file 1 [file cancers-14-05235-s001.zip › cancers-1960505-supplementary.pdf]

Supplementary Materials

Supplementary Table S1: Validation of the differentially expressed genes identified by GATHER using pathway over-representation analysis based on Database for Annotation, Visualization and Integrated Discovery (DAVID – https://david.ncifcrf.gov/).

CAF Marker	**Top 20 differentially expressed genes**	**The most significant over-represented KEGG pathway**	**P-value**
COL11A1	**High CAF vs. Medium CAF**	Protein digestion and absorption	<0.001
	**Medium CAF vs. Low CAF**	ECM-receptor interaction	<0.001
S100A4	**High CAF vs. Medium CAF**	ECM-receptor interaction	<0.001
	**Medium CAF vs. Low CAF**	Protein digestion and absorption	<0.001
CXCL12	**High CAF vs. Medium CAF**	Protein digestion and absorption	<0.001
	**Medium CAF vs. Low CAF**	Focal adhesion	<0.001
C3	**High CAF vs. Medium CAF**	Lysosome	<0.001
	**Medium CAF vs. Low CAF**	Protein digestion and absorption	<0.001
FBLN1	**High CAF vs. Medium CAF**	PI3K-Akt signaling	<0.001
	**Medium CAF vs. Low CAF**	Focal adhesion	<0.001

Supplementary Table S2: Top 10 most significant differentially expressed genes in the dataset of the present study identified using two common RNA-seq data analysis methods such as edgeR and DESeq2.

**Methods**	**edgeR**	**DESeq2**
**G1**	*TMSB10*	*COL3A1*
**G2**	*COL3A1*	*FTL*
**G3**	*FN1*	*SPARC*
**G4**	*SPARC*	*TMSB10*
**G5**	*FTL*	*POSTN*
**G6**	*POSTN*	*FN1*
**G7**	*ACTB*	*LUM*
**G8**	*TMSB4X*	*VIM*
**G9**	*B2M*	*LGALS1*
**G10**	*LUM*	*B2M*

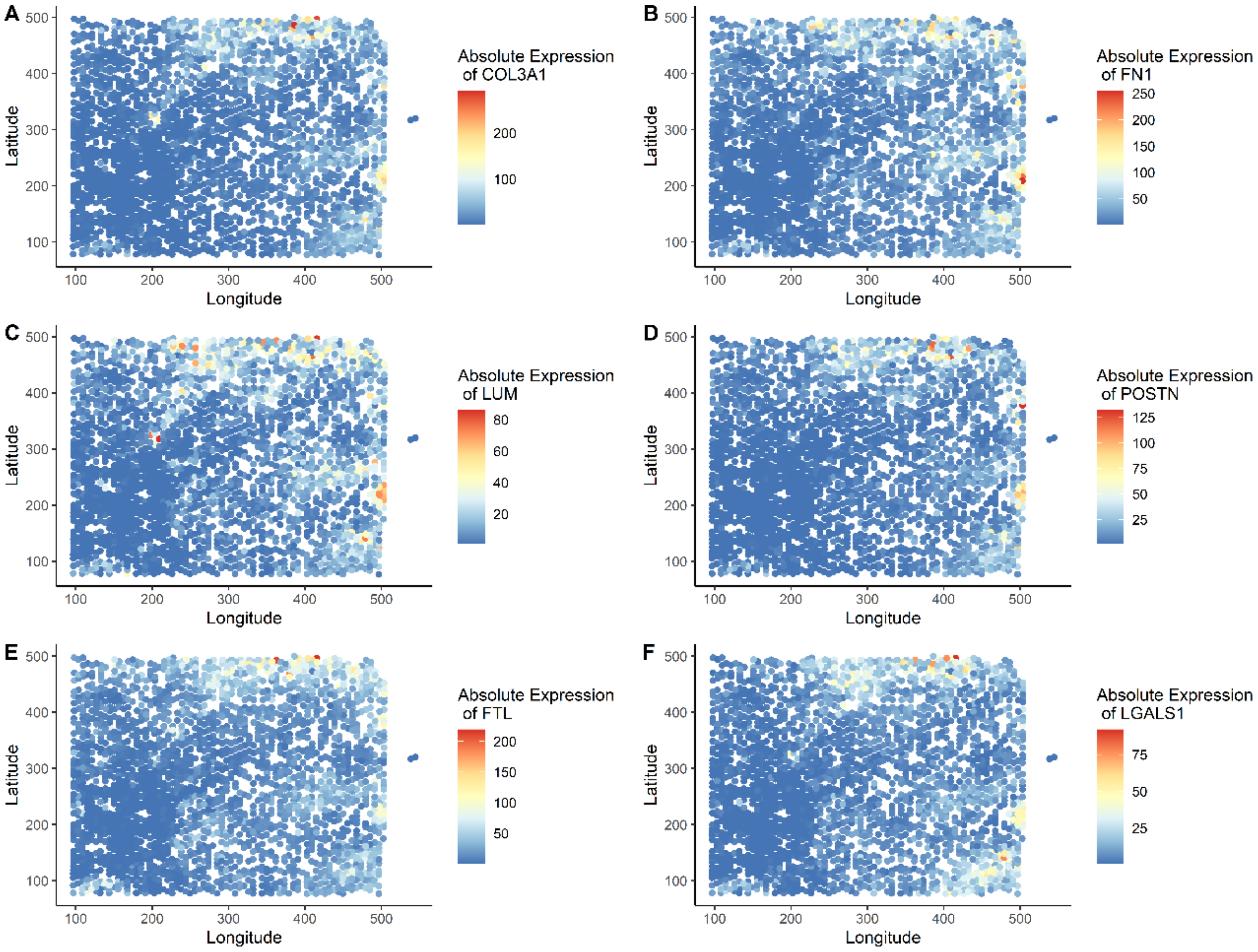

Supplementary Figure S1: The spatial distribution of six selected genes from the supplementary table T2 are shown. Longitude and latitude show the spatial coordinates of cells in the input tumor sample, and colors show the absolute expression of each of the selected genes over the tissue space. Red/blue color indicates high/low expression of the gene in a given cell.
